# All things considered, my risk for diabetes is medium: A risk personalization process of familial risk for type 2 diabetes

**DOI:** 10.1111/hex.12986

**Published:** 2019-10-23

**Authors:** Sandra Daack‐Hirsch, Lisa L. Shah, Kaitlyn Jones, Brenda Rocha, Megan Doerr, Emily Gabitzsch, Thad Meese

**Affiliations:** ^1^ University of Iowa College of Nursing Iowa City Iowa; ^2^ Cleveland Clinic Cleveland Ohio

**Keywords:** mixed methods, risk perception, risk personalization process, salience, type 2 diabetes, vulnerability

## Abstract

**Background:**

A positive family history of type 2 diabetes (T2D) has been associated with risk awareness and risk‐reducing behaviours among the unaffected relatives. Yet, little is known about how people with a positive family history for diabetes develop and manage their personal sense of risk.

**Objective:**

To characterize two key concepts, salience and vulnerability, within the familial risk perception (FRP) model among unaffected individuals, at increased familial risk for T2D.

**Design:**

We conducted a mixed method study. Descriptions of salience and vulnerability were collected through semi‐structured interviews. Participant's perception of self‐reported risk factors (family history, age, race/ethnicity, medical history, weight and exercise) was measured using the *Perceived Risk Factors for T2D Tool* and was compared to a clinical evaluation of the same risk factors.

**Results:**

We identified two components of salience: (a) concern for developing T2D and (b) risk awareness triggers, and two features of vulnerability: (a) statement of risk and (b) risk assessment devices. Although few participants (26%) were concordant between their perceived and clinical overall T2D risk, concordance for individual risk factors was higher, ranging from 42% (medical history) to 90% (family history).

**Discussion and conclusion:**

Both familial and non‐familial events lead people to contemplate their T2D risk, even among people who have a positive family history. Participants often downplayed their overall risk and underestimated their overall risk compared to a clinical risk assessment of the same self‐reported risk factors. Clinicians could leverage key components of the FRP process as way to engage patients in risk reduction strategies earlier.

## 1 INTRODUCTION

The systematic collection of a family medical history captures information about shared inherited, environmental and behavioural risk factors for genomically complex diseases such as cancer, cardiovascular disease and diabetes. Goals for eliciting a family history include risk classification for the purposes of early detection and counselling to support behaviour changes to prevent the disease and/or minimize health complications related to these diseases.^1^ Additionally, a positive family history of T2D has been associated with developing risk awareness and engaging in risk‐reducing behaviours among the unaffected relatives.^2^, ^3^, ^4^, ^5^, ^6^ Yet, little is known about how people with a positive family history for complex diseases such as diabetes develop and manage their personal sense of risk.^7^ Understanding this process could facilitate better collaboration between health‐care providers and patients aimed at prevention and risk reduction interventions.^7^

**Figure 1:**
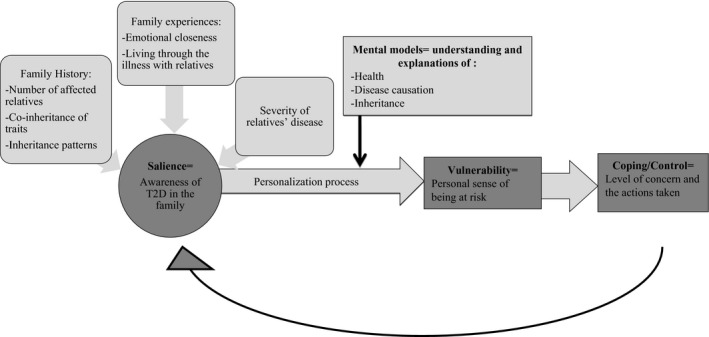
Familial risk perception (FRP) personalization model

To that end, Walter et al^8^ developed the familial risk perception (FRP) personalization model. The model is comprised of four major constructs: salience, mental models, vulnerability and coping/control. Walter et al^8^, ^9^ posit that the FRP personalization process is initiated among unaffected family members when a family member is diagnosed. In FRP, the risk personalization process involves a coalescence of salience (sense of awareness of family history, experiences and disease severity), personal mental models of health (explanations of disease causation and inheritance) and notions of how alike one is to their affected family members. In turn, these factors influence a person's sense of risk and vulnerability for developing the disease. The level of perceived risk influences coping and risk control strategies, which may or may not include behaviour changes (Figure 1).^9^ The original model was largely drawn from cases of familial cancer and coronary artery disease with very few examples of diabetes.^8^, ^9^

Thus, we planned a mixed methods study to further develop the FRP model for people with a positive family history of T2D and are, themselves, unaffected. Previously, we described beliefs about cause, genetics and inheritance for T2D among participants in this study.^10^ In this article, we characterize salience and vulnerability. We had two research questions: (a) how do people at increased familial risk for T2D describe salience and vulnerability (qualitative arm) and (b) how does perceived diabetes risk compare to a clinical assessment of diabetes risk among individuals at increased familial risk for T2D (quantitative arm).

## 2 METHODS

### 2.1 Overview of Core Mixed Method Study

**Figure 2:**
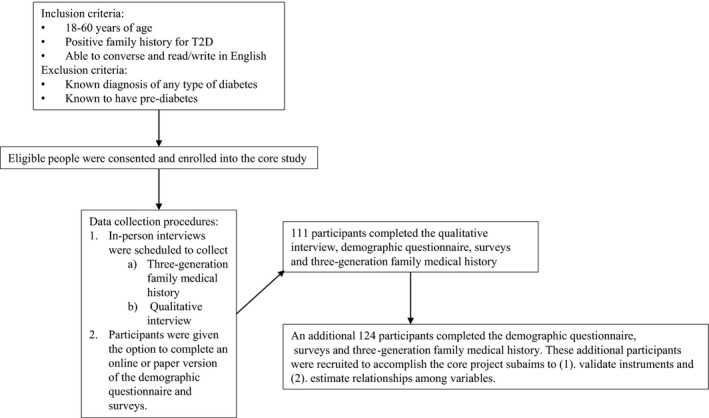
Core study participant enrolment and study flow

To further develop the FRP model for T2D, we conducted a mixed method study using a concurrent design.^11^ In this type of mixed method approach, data are collected simultaneously. We selected this design because it allows investigators to elucidate complementary aspects of the same phenomenon and can facilitate a deeper understanding of participants’ responses. The qualitative arm was the primary focus of our core project. We used a single semi‐structured interview to collect data on the FRP model domains. In the quantitative arm, we collected supplemental information about each domain through a survey. Figure 2 provides an overview of participant enrolment and study flow.

### 2.2 Participant Recruitment

The study took place in the United States, Midwest in two locations with populations of 450 000 (comprised of urban and rural communities) and 27 000 (single urban community), respectively. All participants were recruited into the core study as follows. Study recruitment posters and brochures were placed in neighbourhood restaurants, hair salons, grocery stores and a community‐based agency that serves lower‐income individuals. We also recruited through a rural, primary care clinic in a largely Hispanic neighbourhood and by mass emails sent to a college campus community. Interested participants directly contacted the research team. Interested individuals from the community‐based organization provided contact information to their case worker, who passed the information to the research team. A research team member screened, obtained consent and enrolled the participants. Inclusion and exclusion criteria are listed in Figure 2. The study was approved by the first author's Institutional Review Board. Specific enrolment issues and data collection, analysis and results with respect to the salience and vulnerability domains of the FRP model are presented for each arm. The results of each arm are interfaced in the discussion.

## 3 QUALITATIVE ARM: THE FRP PERSONALIZATION PROCESS

### 3.1 Participant enrolment goals

Because one of the major goals of the core study was to identify subtypes of the FRP personalization process that combined qualitative and quantitative data using qualitative cluster analysis, we enrolled a larger number of participants than typical for a qualitative study. Based on previous qualitative studies using cluster analysis, we determined at least 100 interviews would be needed.^12^, ^13^ To achieve diversity and ability to conduct the cluster analysis, our goal was to enrol 30 participants in each non‐Hispanic White, non‐Hispanic Black and Hispanic groups. Although individuals from other ethnic groups were not excluded, very few people from other ethnicities lived in the study's catchment area.^10^

### 3.2 Data collection

We conducted a semi‐structured interview based on Walter and Emery's^9^ original study (refer to Table 1 for the interview guide) and collected a three‐generation family health history focusing on any type of diabetes and metabolic syndrome. The interviews took place in person or over the phone and lasted between 30 and 90 minutes. Each interview was audio‐recorded and transcribed verbatim, and then uploaded into NVivo 10.^14^ The family histories were recorded and stored in Progeny.^15^ After completing the interview, participants received a $25 gift card.^10^

**Table 1:** Interview guide for salience and vulnerability domains

Domain	Interview questions
Salience	Do you ever think about your risk? What makes you think about your risk? When do you think about it? What makes getting diabetes matter to you?
Vulnerability	What do you think makes you prone to T2D? How would you rate your risk for T2D? o Why is that?

### 3.3 Data analysis

All transcripts were analysed using direct content analysis, a deductive process most appropriate for validating or extending an existing conceptual framework.^16^ Qualitative coding was conducted in three stages.^10^ Stage one consisted of reading each transcript and creating a narrative summary of first impressions. Stage two consisted of coding all text that represented topics related to one of the major domains of the FRP model (Salience, Mental Models—disease causation/health and inheritance, Vulnerability, and Coping and Control). In stage three, we executed a series of data extractions from NVivo by aggregating data from each of the major domains and identified subcategories that represented nuances of each. All coding was conducted by at least two investigators. Coding discrepancies were discussed, and unresolved discrepancies were brought to the larger research team for clarification and comparison to the narrative summaries created in stage one. When consensus could not be reached, a new code was developed. Topics outside the FRP framework were identified and analysed to determine whether they represented a new category or a subcategory of an existing code. The results presented in this article are specific to the FRP model domains of salience and vulnerability.

## 4 RESULTS

We summarized participant characteristics in Table 2. Just over half of the participants identified as female (n = 61, 55%), and over half reported their race as something other than non‐Hispanic White (n = 64, 58%). Generally, the study participants were young and well‐educated. Three people did not have a first‐degree relative (FDR) with diabetes. However, their family history was consistent with metabolic syndrome and therefore high risk for T2D, or in one case a maternal grandmother had T2D and had raised the participant.

**Table 2:** Demographics by ethnicity N = 111

Demographic/group n (%)	Asian n = 13 (12)	Hispanic n = 28 (26)	Non‐Hispanic Black n = 19 (17)	Non‐Hispanic White n = 47 (42)	Other n = 4 (3)	Total sample summary (N = 111)
Age (y)						
Range	19‐39	18‐46	18‐42	18‐47	25‐35	18‐47
Mean (SD)	30 (6.9)	27 (7.4)	29 (7.3)	29 (5.7)	32 (4.5)	29 (6.5)
Familial Risk Category n (%)^a^						
Average	0	0	3 (16)	0	0	3 (3)
Moderate	5 (38)	11 (39)	8 (42)	26 (55)	1 (25)	51 (46)
High	8 (62)	17 (61)	8 (42)	21 (45)	3 (75)	57 (51)
Gender n (%)						
Female	6 (46)	17 (61)	12 (63)	24 (51)	2 (50)	61 (55)
Male	7 (54)	11 (39)	7 (37)	23 (49)	2 (50)	50 (45)
Education n (%)						
High school or less	0	1 (4)	1 (6)	1 (2)	0	3 (3)
Some college	2 (15)	13 (46)	5 (29)	6 (13)	0	26 (24)
2‐ or 4‐year college degree	2 (15)	8 (29)	5 (29)	20 (43)	3 (75)	38 (35)
Graduate or Professional degree	9 (70)	6 (21)	6 (35)	19 (41)	1 (25)	41 (38)
			2 (no report)	1 (no report)		3 (no report)
Marital status n (%)						
Married/Partnered	7 (54)	9 (33)	2 (12)	25 (54)	0	43 (41)
Single, Separated/ Divorced	6 (46)	18 (67)	15 (88)	21 (47)	3 (100)	63 (59)
		1 (no report)	2 (no report)	1 (no report)	1 (no report)	5 (no report)
Income n (%)						
<10k	2 (15)	5 (18)	3 (18)	9 (20)	1 (25)	20 (19)
10k‐49k	6 (46)	19 (68)	12 (70)	10 (21)	2 (50)	49 (45)
50k‐99k	3 (23)	2 (7)	1 (6)	19 (42)	1 (25)	26 (24)
>100k	2 (15)	2 (7)	1 (6)	8 (17)	0	13 (12)
			2 (no report)	1 (no report)		3 (no report)

a *Average*: Only: (a) 1 second‐degree relative (SDR) with diabetes from one or both sides, or; (b) No family history. *Moderate*: Only: (a) 1 first‐degree relative (FDR) with diabetes, (b) 1 FDR and 1 SDR with diabetes from the same lineage, or (c) 2 SDR from the same linage with diabetes. *High*: At least: (a) 2 FDR, (b) 1 FDR and 2 SDR with diabetes from the same lineage, (c) 3 SDR with diabetes from the same lineage, or (d) ‘Moderate risk’ family history on both sides of pedigree^2^, ^45^ (reproduced QHR^10^).

Table 3 includes a summary of thematic categories and subcategories for the salience and vulnerability domains. Narrative descriptions are provided below.

**Table 3:** Summary of thematic categories and subcategories by domain

FRP domain	Thematic category	
Salience	Developing T2D is concerning because…	Consequences of T2D are serious Mortality/longevity Burden—personal and financial Generally health conscientious Non‐familial exposures to diabetes information Desire to prevent T2D in other family members
	Risk awareness triggers	Personal milestone or life event (eg birthday and diagnosis of family member) Awareness of risk factors (other than family history) Family history Severity of relative's disease Caring for sick relative Formal or informal educational experiences
	T2D is a manageable disease	
	Personal risk factors	Behaviours Family history Weight Age Race/ethnicity Sex Gestational diabetes Asymptomatic for T2D Knowledgeable about T2D
Vulnerability	Risk perception	Low Medium High

### 4.1 Salience

Only five participants reported they really did not think about their risk. Twenty‐one participants reported that although not a burning issue, T2D risk is ‘always in the back of their mind’. The majority (n = 85) felt that T2D risk was a major concern in their life. We identified two over‐arching components of salience: (1) concern for developing T2D and (2) risk awareness triggers.

#### 4.1.1 Developing T2D is concerning

Part of salience is developing a heightened sense of concern—that T2D is a serious disease. Central themes included a diagnosis of T2D is burdensome, has serious consequences and could shorten life expectancy. Participants reported that T2D can be a financial burden and it can lead to additional self‐care activities, restrictions on foods and beverages that people enjoy, comorbidities and other health problems, and a diminished quality of life as depicted in the following quotes:

You know you have a little more freedom when you don’t have that problem. And once you have diabetes, I mean you have to watch yourself so strictly. There’s a lot of things you can't do. And you can be at risk to lose your foot or all kinds of things once you have [T2D].

Participants also described non‐familial exposures to information and situations about T2D that gave them pause. These two individuals recalled media and work experiences:

But, you know it’s all over the news now. I mean it seems like every other day you turn on the TV and there’s commercials about it, or I read a few health magazines and it seems like there is usually an article that mentions it or whatever, so I mean *it’s a big deal.*

I work in an inpatient mental health care facility, part of that is a residential care facility where people not only have schizophrenia or other mental health issues but also severe physical problems. I can’t tell you the number of people who have diabetes—both clients and staff… it makes me sad. *I don’t want that to be me*.

Diabetes was not necessarily participants’ primary concern. For some, overall health and disease prevention in general were key:

I think more for [my] personal sake you know, having that health‐conscious kind of mind set and… that [diseases] can be avoided. I don’t want any disease.

A few participants felt that it was their duty to take care of themselves and set a good example for other family members so that T2D could be prevented.

I want to stop that from happening to my kids too. I want them to have a better example of the [healthy] lifestyles. I want them to have an example, what I mean is my dad is diabetic, and then I will be diabetic, and then my kids will say oh god, we will have diabetes too! So, I want to stop it here.

However, some participants’ concern about developing T2D was moderated by the idea that T2D is a manageable disease and not as life‐threatening as cardiac diseases and cancer.

People get cancer, like all the time. And it kills them. And people get diabetes all the time, but it doesn’t seem to kill them. Diabetes it’s more like, they manage it. I don’t see diabetes as being fatal.

#### 4.1.2 Risk awareness triggers

Family characteristics such as a positive family history, a personal experience with a family member who has T2D and the severity of a family member's disease prompted participants to think about their own risk. A positive family history was most often cited. However, the family history was not always specific to diabetes. For example, excessive weight and lifestyle habits that perpetuated in a family were also considered.

Uh, well, so I’m aware that… the people in my family who have type 2 diabetes, after putting on a few pounds or an illness or whatever and continued Western lifestyle, sitting at a desk…you know, ended up with type 2 diabetes.

Personal experiences with affected family members centred on perceptions about how well the affected family member was managing their diabetes. For some, having gone through diabetes management classes with their family members was a significant ‘wake‐up call’. About 20% (n = 23) said it was not until their loved one had a serious complication (amputation, heart attack, blindness, extended and emergency hospitalization) that they began to think about their own risk.

Events, personal milestones and awareness of specific personal risk factors were also instrumental in participants’ development of salience. Of interest were individuals who recalled poignant events when their risk was made unambiguous to them. These events included an affected family member saying, ‘you are at risk’, sharing the news of their diagnosis or being recently hospitalized for complications related to T2D or other diseases. However, events were not necessarily family related as exemplified here:

Lately, I’ve been thinking about [my risk]. Um, it’s been about a year, my cat [laughs], my cat died, and he had diabetes.

I was rejected [as a sperm donor] because both my mother and both her parents um, type 2 diabetes. *Interviewer:* So was that the first time you really thought about your own risk for diabetes? *Participant*: Yep

Personal milestones also triggered salience. For example, some participants had recently turned or were nearing an age they considered to signify increased risk, namely between the ages of 30 and 45 years or the age at which their relative was diagnosed. Others talked about becoming an adult and more responsible for their lifestyle and health. Having a family of one's own and being exposed to content about diabetes in formal and informal educational settings also helped to create awareness of T2D risk.

Awareness of personal risk factors largely had to do with current lifestyle habits—specifically diet and exercise. For example, participants passed judgement on how well they managed diet and exercise, and some reflected that in statements about their cholesterol and weight. For example, when weight and cholesterol were high and lifestyles were judged to be poor, participants thought about their risk for T2D.

### 4.2 Vulnerability

We identified two features of vulnerability: (a) statement of risk perception and (b) risk assessment devices. Risk appraisal was collected by directly asking participants how they would rate their personal risk for diabetes (prompted as needed by asking participants if their risk was high, medium, low or something else). Responses were fairly equally divided into high (n = 31; 30%), medium (n = 41; 37%) and low (n = 39; 35%). If participants had not spontaneously described how they came to their stated risk, we asked participants to elaborate. In doing so, participants named personal risk factors that either increase or decrease their risk and verbalized a rationale for the stated risk, which we categorized into different assessment devices.

#### 4.2.1 Risk assessment devices

Risk assessment devices are cognitive strategies people used to explain their risk value. Participants generally took several risk factors into consideration when assessing their personal risk. Only two participants said family history alone accounted for their ‘high’ risk to develop T2D. During the interview, participants often had an intrapersonal dialog where they judged how well they were adhering to exercise/activity levels and dietary habits and managing their weight as justification for their stated risk given uncontrollable risk factors (genetics/family history, sex and race). We called this counterbalancing of risk factors. For example, this participant believes her risk to be low despite the high familial risk rank because she has ‘counterbalanced’ her familial risk against behaviour changes: ‘Right now I think my risk would be low because I work out, avoid coffee and the sugary good things. So, I think its low, but it's me who's keeping it that way’. Risk factors other than family history could also offset each other. For example, a less‐than‐ideal lifestyle could be tolerated at a younger age, so age neutralized lifestyle choices. Others said they were not adhering to healthy lifestyle behaviours or added risk factors up to rationalize their stated risk.

Participants also compared themselves with family members, non‐family members and past versions of themselves to rationalize their risk assessment. Comparisons between their own and others (or past self) body type (eg pear‐shaped, central obesity), lifestyles and behaviours (*‘I have a sweet tooth like my dad’*) and age at diagnosis were commonly made when formulating personal risk.

A small subset of participants (n = 7) did not base their risk on a self‐assessment of personal factors. Rather, they expressed being put on notice by a health‐care professional or family member who made it explicit, ‘you are at increased risk’ and this message was internalized.

### 4.3 Quantitative arm: comparison of clinical risk assessment and perceived risk for T2D

To further characterize salience and vulnerability in the FRP, we also compared participants’ perceptions about individual risk factors and overall risk to a clinical assessment of individual risk factors and overall risk for T2D. For this analysis, we identified a subset of participants from the core study who completed assessments of both perceived and clinical measures of risk for T2D (n = 153).

### 4.4 Data collection

**Figure 3:**
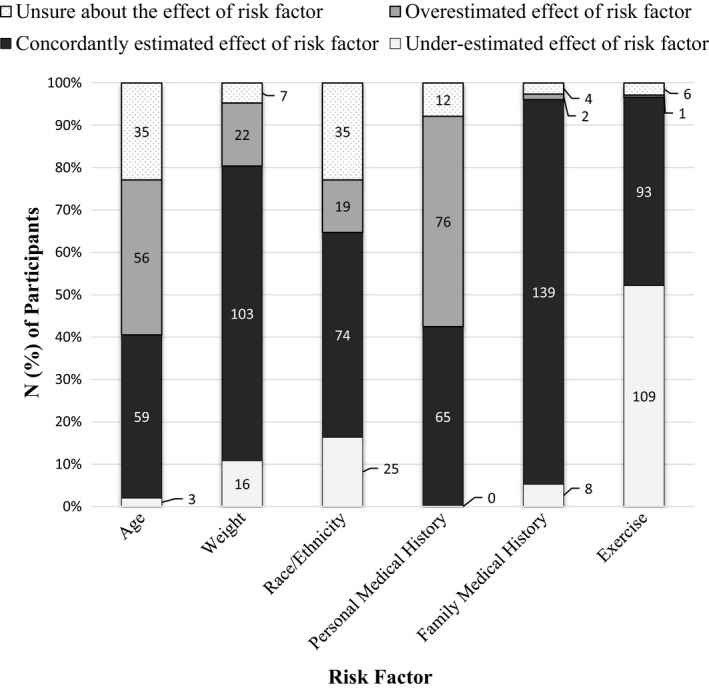
Comparison of perceived risk factors and clinical risk factors

We collected perceived and clinical measures of risk for T2D as part of a survey (refer to Figure 3). After completing the survey, participants received a $25 gift card. Study data were collected and managed using REDCap electronic data capture tools^17^ hosted at The University of Iowa.

### 4.5 Instruments

#### 4.5.1 Perceived risk factors for type 2 diabetes (PRF‐T2DM)

The PRF‐T2DM is a measure of perceived personal risk for T2D.^18^ Using a four‐point Likert scale (0 = I do not know, 1 = there is no effect on risk, 2 = decreases the risk, 3 = increases the risk), participants rated the effects of each of 12 risk factors on their risk for developing T2D. Participants assessed the following risk factors: age, weight, race/ethnicity, personal medical history, family medical history, diet habits, exercise habits, financial resources, support resources, neighbourhood resources, community resources and work/school conditions. The sum of the responses makes up the total score, and higher scores represent heightened perception of risk factors (salience). PRF‐T2DM has high internal consistency (0.81) and reliability (0.83) based on major risk factors for T2D, demonstrating construct validity.^18^ We found the overall internal reliability (α = 0.68) and validity of the PRF‐T2DM to be acceptable in our study population.^19^ Perception of overall risk for diabetes (vulnerability) was assessed by asking ‘What is your overall risk to develop type 2 diabetes?’ Participants rated their overall risk as no risk, low risk, moderate risk or high risk.^18^ This question was placed immediately following the 12 risk factors on the PRF‐T2DM.^19^

#### 4.5.2 Clinical risk assessment

Cleveland Clinic developed MyFamily, a patient‐centred—family health history collection and clinical decision support tool. MyFamily classifies individuals into risk categories based on age, weight/BMI, race/ethnicity, personal medical history, personal history of gestational diabetes and family history of T2D. For this study, the medical history portion consisted of a self‐reported health status questionnaire to elicit personal history of cardiovascular disease and treatment, cholesterol and triglyceride levels, and use of antipsychotics and glucocorticoid medication. Participants selected their height and weight from a drop‐down list containing height in centimetres and feet and inches, and weight in kilograms and pounds; BMI was calculated as weight(kg)/height^2^(m^2^) and participants were classified as underweight (BMI ≤ 18.5), normal (BMI = 18.5‐24.9), overweight (BMI = 25.0‐29.9) or obese (BMI ≥ 30.0). Overall, clinical risk algorithms were developed by teams of clinician experts using a standardized process and templates informed by the Institute of Medicine's report *Clinical Practice Guidelines We Can Trust*.^20^ Clinical guidelines and primary literature were gathered and translated into a consistent ontology for inter‐comparison; statements were selected for inclusion in the risk assessment based on clinical evidence and, where evidence was lacking, consensus opinion of the clinician expert team. Multipart (compound) Boolean statements were used to assess risk in the T2D algorithm and resulted in classification of participant's overall risk for diabetes as population risk (low risk), double the population risk (moderate risk) or triple the population risk (high risk). In other words, the algorithm was designed to identify the level or presence (or absence) of risk factors known to play a role in T2D risk and assign a risk category based on clinical evidence and expert opinion.

#### 4.5.3 Exercise

We also collected data on activity levels using the International Physical Activity Questionnaire (IPAQ)^21^ because the Cleveland Clinic MyFamily does not include activity level. IPAQ is comprised of 4 questionnaires to measure the duration, frequency and intensity of past week physical activity within leisure, transportation, occupational and domestic domains of physical activity in persons aged 15‐69 years. Its psychometric properties have been extensively established.^21^, ^22^ For this analysis, we used only the leisure physical activity subscale because 150 minutes of moderate or vigorous activity per week is recommended to reduce risk for T2D.^23^ We summed the total minutes of moderate and vigorous leisure exercise and created a binary variable. Those reporting 150 minutes of moderate to vigorous exercise per week were classified as exercise is ‘not increasing risk’ and those with less than 150 minutes of moderate to vigorous exercise per week classified as exercise is ‘increasing risk.’

### 4.6 Analysis

Data were exported from REDCap into SAS 5.1 for analysis. Based on a comparison of their perceived overall risk and their clinical overall risk (risk calculated by the MyFamily algorithm), participants were classified into one of three overall risk perception groups: (a) ‘under‐estimators’ if perceived risk< clinical risk, (b) ‘concordant‐estimators’ if perceived risk = clinical risk or (c) ‘over‐estimators’ if perceived risk > clinical risk. Demographics were calculated for each of these three groups, including medians and interquartile range for continuous non‐parametric variables and frequency counts and percentages for categorical variables. Comparisons of demographics among these groups were calculated using a Wilcoxon test for age and numeracy, and Fisher's exact test for sex, race/ethnicity, highest education completed, marital status and BMI classification.

We also compared perceived risk and clinical risk estimates on six individual risk factors: age, weight, race/ethnicity, personal medical history, family medical history and exercise habits. These six risk factors were selected because assessments of the effects of these risk factors on diabetes risk were available on both tools for comparison and these risk factors were also reported by participants in the qualitative interviews. For each of the six risk factors, participants were classified into one of four risk‐factor perception groups based on a comparison between their perception of the effect of that risk factor (PRF‐T2DM) and a clinical assessment of the effect of that risk factor (MyFamily plus IPAQ for exercise). The risk‐factor perception groups were (a) underestimate effect of risk factor, (b) concordantly estimate effect of risk factor, (c) overestimate effect of risk factor and (d) did not know effect of risk factor and were created, as described in Table 4.

**Table 4:** Individual risk factor perception classifications

Risk Factor Perception Group		Participants’ rating of individual risk factor effect (PRF‐T2DM)		Clinical estimate of individual risk factor effect (MyFamily and IPAQ)
Underestimate effect of risk factor	if	Risk factor: Decreases risk or There is no effect on risk	and	Risk factor increases risk
Concordantly estimate effect of risk factor	if	Risk factor: Increases risk	and	Risk factor increases risk
	if	Risk factor: Decreases risk or There is no effect on risk	and	Risk factor does not increase risk
Overestimate effect of risk factor	if	Risk factor: Increases risk	and	Risk factor does not increase risk
Did not know effect of risk factor	if	Risk factor: Do not know the effect	and	Risk factor: increases risk or does not increase risk

The four perceived risk‐factor groups were stratified by overall risk perception (under‐, concordant‐ or over‐estimators). Frequencies were calculated for each risk factor (age, weight/BMI, race/ethnicity, personal medical history, family medical history, exercise habits) for each stratified group. Fisher's exact test was used to compare how each risk factor was perceived between those who underestimated their overall risk and those who concordantly estimated their overall risk.

## 5 RESULTS

These analyses were conducted on 153 participants who completed the PRF‐T2DM, health status questionnaire and the IPAQ. Fifty‐six of the 153 had also completed a qualitative interview.

### 5.1 Overall risk

Most participants (n = 113; 74%) were discordant between their perceived overall risk and their clinical overall risk; most underestimated their risk, and only two perceived their overall risk for diabetes as higher than their clinical overall risk. Forty participants (26%) were concordant between their perceived overall risk and their clinical overall risk for diabetes. Of these 40 participants, 12 were at moderate risk for diabetes and 28 were at high risk. The only demographic difference among the three groups was BMI classification. There was near significance in meeting exercise requirements between the three groups. There were no differences in age, numeracy, sex, race/ethnicity, education or marital status among the three groups (Table 5).

**Table 5:** Demographics Comparison among Participants

Characteristic	Participants’ Perceived Risk Compared to Clinical Risk					
	‘Under‐Estimators’ *Perceived Risk < Clinical Risk*		‘Concordant‐Estimators’ *Perceived Risk = Clinical Risk*		‘Over‐Estimators’ *Perceived Risk > Clinical Risk*	
	n = 111 (73)		n = 40 (26)		n = 2 (1)	
	Median (min, max)	IQR (Q1, Q3)	Median (min, max)	IQR (Q1, Q3)	Median (min, max)	IQR (Q1, Q3)
Age, y (*P* = .56)	32 (18, 60)	13 (26, 39)	30.5 (19, 59)	12.5 (24, 36.5)	27 (26, 28)	2 (26, 28)
Numeracy score^a^ (*P *= .08)	5 (0, 6)	2 (4, 6)	5 (2, 6)	2 (4, 6)	1.5 (0, 3)	3 (0, 3)
Characteristic, N (% of row)						
Sex (*P* = .58)	n = 111		n = 40		n = 2	
Male	46 (75)		15 (25)		0 (0)	
Female	65 (71)		25 (27)		2 (2)	
Race/ethnicity (*P* = .43)	n = 106		n = 39		n = 2	
Asian	18 (75)		6 (25)		0 (0)	
Hispanic	24 (73)		9 (27)		0 (0)	
Non‐Hispanic Black	17 (65)		7 (27)		2 (8)	
Non‐Hispanic White	47 (73)		17 (27)		0 (0)	
Highest education completed: (*P* = .70)	n = 108		n = 38		n = 1	
High school or less	3 (60)		2 (40)		0 (0)	
Some college	24 (73)		8 (24)		1 (3)	
2‐ or 4‐y college degree	36 (73)		13 (27)		0 (0)	
Graduate or professional degree	45 (75)		15 (25)		0 (0)	
Marital Status (*P* = .34)	n = 105		n = 38		n = 0	
Single, separated, divorced, widowed	56 (70)		24 (30)		0 (0)	
Married/partnered	49 (78)		14 (22)		0 (0)	
Weight classification according to BMI (*P* = .004)	n = 110		n = 39		n = 1	
BMI underweight or Normal Weight	39 (74)		13 (24)		1 (2)	
BMI overweight	43 (88)		6 (13)		0 (0)	
BMI obese	28 (58)		20 (42)		0 (0)	
Exercise classification (*P *= .06)	n = 110		n = 39		n = 2	
<150 min of moderate to vigorous exercise/week	8 (50)		8 (50)		0 (0)	
≥150 min of moderate to vigorous exercise/week	102 (68)		31 (21)		2 (1)	

a Numeracy was assessed with a six‐item numeracy questionnaire that assesses numeracy skills. We combined two, 3‐item questionnaires.^46^, ^47^ The score equals the total number of correctly answered questions.

### 5.2 Individual risk factors

Although just 26% of participants were concordant between their perceived overall risk and clinical overall risk for diabetes, the proportion of participants that were concordant on individual risk factors was higher, ranging from 42% (personal medical history) to 90% (family medical history). Participants most often overestimated the effect of their personal medical history (n = 76; 42%) and most often underestimated the effect of their exercise habits (n = 109; 52%) on their risk for diabetes. Participants’ perceptions of family history (n = 139; 91%), race/ethnicity (n = 74; 48%) and weight (n = 106; 67%) were mostly concordant with the clinical assessment. Figure 3 shows comparisons between PRF‐T2DM and MyFamily for individual risk factors, illustrating the number of participants that overestimated, underestimated, concordantly estimated or were unsure about the effect of six risk factors on their risk for diabetes.

**Figure 4:**
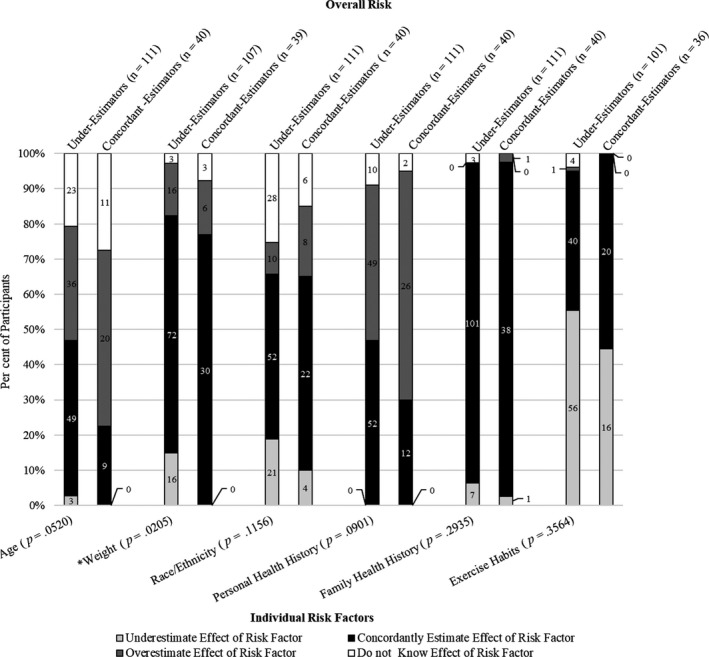
Comparison of risk factors perception between under‐estimators and concordant‐estimators of overall risk

We found that several participants concordantly or overestimated the effect of individual risk factors, yet still underestimated their overall risk for diabetes (Figure 4). The effect of exercise was frequently underestimated by both those who underestimated (n = 56; 55%) or concordantly estimated (n = 16; 44%) their overall risk. These participants felt their exercise habits decreased their risk for diabetes; however, they did not meet the requirement of 150 minutes of moderate to vigorous exercise per week. Weight was the only risk factor that showed a significant difference between those who underestimated overall risk and those who concordantly estimated overall risk (*P* = .0205). Interestingly, none of those who concordantly estimated their overall risk underestimated the effect of their weight on their risk. However, participants in both overall risk groups tended to be concordant on their perception and the clinical assessment of the effect of their weight on overall T2D risk.

## 6 DISCUSSION

Consistent with Walter et al^8^, ^9^ and others^3^, ^24^ we found family characteristics such as a positive family history, a personal experience with a family member who has T2D and the severity of a family member's disease stimulated people to think about their own risk. However, given the major inclusion criteria was a  positive family history, these results are not surprising. We found that in addition to family, non‐familial events and personal milestones that are encountered in and over the course of peoples’ everyday lives pique risk awareness and can lead to concern about developing T2D.

Walter et al^8^, ^9^ defined vulnerability as the outcome of processing the salient features of one's family history and experiences into a sense of personal individual risk. Further, these authors posit the FRP process is an intermittent dynamic process based on on‐going family events. However, our data show that risk perception was based on an interpretation and balancing of individual risk factors that included family history rather than only on‐going family events.

Participants’ expression of vulnerability included identifying and taking into consideration multiple risk factors and was consistent with the public's understanding that T2D is a complex disease.^10^, ^25^ However, their conclusions about the impact of an individual risk factor and/or their overall risk assessment were not always consistent with how these same self‐reported risk factors were assessed using a clinical algorithm (MyFamily). For example, most participants in the quantitative arm underestimated their overall risk for diabetes compared to the MyFamily risk estimate, despite accurately perceiving how age, race and family history affect their risk for T2D.

Optimistic bias could be contributing to the overall underestimation of diabetes risk. This is a cognitive process that leads people to believe they are less likely to suffer from a negative event (ie develop a disease) and more likely to experience a positive outcome than the data suggest.^26^, ^27^, ^28^, ^29^ We believe that participants expressed optimistic bias during qualitative interviews. For example, they seemed to downplay their overall risk, determining their overall risk to be medium or low and justifying their conclusion by comparing themselves to others and past versions of themselves and counterbalancing risk factors (eg although family history increased risk, exercise may be viewed as reducing risk; therefore, overall risk is estimated to be medium). The possibility of optimistic bias is also supported by the quantitative data when participants correctly perceived relevant risk factors as significant to their overall risk but judged their overall risk to be lower compared to the clinical risk algorithm (MyFamily). Misperceptions about the impact of individual risk factors on their overall risk also support the possibility of optimistic bias (eg, perceiving exercise to lower risk and self‐reporting less than 150 minutes of moderate to vigorous exercise per week). Dickerson et al^28^ found that college students, similar in age to this study's participants, minimized their perceived overall risk for diabetes by downplaying the effect of lifestyle factors and basing their risk more heavily on non‐controllable factors.

### 6.1 Applicability of the findings

Both provider and patient explanations of health and risk for disease provide a clinical reality, and divergence between clinician and patient explanations of these processes can impede patients’ uptake of healthful behaviours.^30^, ^31^ Health‐care professionals understand risk from a technical and statistical perspective, while patients may have a more experiential, personal and affective risk perspective.^32^, ^33^, ^34^, ^35^ However, clinicians can influence patients’ risk perception adjustments.^4^, ^36^, ^37^ As such, this study involved a relatively young group of participants, and key events that led to salience about T2D were reported prior to being diagnosed as pre‐diabetic or with T2D.

Given our findings, we assert conversations about T2D risk do not happen soon enough or often enough. If misperceptions about risk persist, patients may naturally bias their risk assessment towards explanations of risk that reinforce their perspective and then delay engaging in risk reduction behaviours.^38^, ^39^, ^40^ The act of taking a family history can be a catalyst for the FRP personalization process and if done earlier it could be leveraged on more proximal salient events before risk perception become engrained. By purposely guiding patients through the FRP personalization process, clinicians and patients could collaboratively identify patients’ T2D risk and develop tailored risk reduction strategies earlier. In this way, the family history can be a tremendously advantageous tool for risk stratification as well as an intervention tool.^2^, ^41^, ^42^, ^43^, ^44^

### 6.2 Limitations

The MyFamily T2D risk assessment algorithm, like many clinical risk evaluation tools, relies on self‐report, so risk estimations are only as good as the information supplied. Risk perception is also dynamic; as such, participants’ perceptions of risk are subject to change. Regardless, our findings showed high discordance of T2D risk estimates between patients’ perception and a clinical tool. Even when participants’ perceived risk is concordant with a clinical risk assessment, the process through which this conclusion is reached may vary. Family history was rarely underestimated (or overestimated) as a risk factor. However, the study was not inclusive of people with low familial risk.

## 7 CONCLUSION

Findings from this study improve our understanding of how people personalize and process their risk for T2D and provide important insight as to when and how ideas of risk are forming and when clinicians could collaborate with patients in this process. More research is needed to understand the relationship between how people process risk and their engagement in actions to mitigate risk.

## CONFLICT OF INTEREST

The authors declare that they have no competing interests.

## Data Availability

The data that support the findings of this study are available on request from the corresponding author. The data are not publicly available due to privacy or ethical restrictions.
